# Evaluation of primary care responsiveness by people with mental illness in Spain

**DOI:** 10.1186/s12913-022-07516-2

**Published:** 2022-01-31

**Authors:** Valle Coronado-Vázquez, María Josefa Gil-de-Gómez, Eva Rodríguez-Eguizábal, Bárbara Oliván-Blázquez, Juan Gómez-Salgado, Rosa Magallón-Botaya, María Antonia Sánchez-Calavera

**Affiliations:** 1 Aragonese Institute for Health Sciences (IACS), 50009 Zaragoza, Spain; 2 School of Medicine, Universidad Francisco de Vitoria, 28223 Madrid, Spain; 3 Illescas Primary Care Health Center, Castilla-La Mancha Health Service, 45200 Toledo, Spain; 4 Group B21-20R, Health Research Institute of Aragon (IIS), 50009 Zaragoza, Spain; 5 redIAPP group RD16/0007/0005, Aragonese Primary Care Research Group, 50009 Zaragoza, Spain; 6 San Pedro Hospital, La Rioja Health Service, 26006 Logroño, Spain; 7 Puerta de Arnedo Primary Care Health Center, La Rioja Health Service, 26580 Arnedo, Spain; 8 University of Zaragoza, 50009 Zaragoza, Spain; 9 Department of Sociology, Social Work and Public Health. Campus El Carmen, Universidad de Huelva, Av. de 3 de Marzo, 21007 Huelva, Spain; 10 Safety and Health Postgraduate Programme, Universidad Espíritu Santo, 092301 Guayaquil, Ecuador; 11 Arrabal Primary Care Health Center, Aragon Health Service, 50009 Zaragoza, Spain; 12 Fuentes Norte Primary Care Health Center, Aragon Health Service, 50002 Zaragoza, Spain

**Keywords:** Responsiveness, Primary care, Mental illness

## Abstract

**Background:**

The health system responsiveness is a concept developed by the World Health Organization that measures patients’ expectations for the non-medical care they receive. The aim of this study is to assess primary care responsiveness as seen by people with mental illness and to analyse the factors associated with poor responsiveness.

**Methods:**

Cross-sectional descriptive study on 426 people with mental illness who had attended primary care consultations at least once in the previous 12 months. The responsiveness of the health system was determined through the short questionnaire “Multi-country Survey Study on Health and Health Systems Responsiveness”. Differences in responsiveness by sociodemographic characteristics were compared through the Chi-squared test. Logistic regression identified the factors associated with poor responsiveness.

**Results:**

Overall responsiveness was measured as good by 77.4% of patients, being this probability higher in the domains: dignity, confidentiality, and communication. The most valued domains by people with mental illness were prompt attention (42.4%), dignity (30.1%), and communication (17%). Only prompt attention scored high importance and poor responsiveness. In patients with an income lower than 900 euros per month and low level of studies, the probability of poor confidentiality responsiveness was multiplied by 3 and 2.7 respectively.

**Conclusions:**

People with mental illness perceive good responsiveness from primary care in terms of dignity, confidentiality, and communication. Prompt attention, as the domain of greatest importance and worst valuation, should be prioritised through the implementation of organisational measures in health centres to reduce waiting times, especially in urban areas.

**Supplementary Information:**

The online version contains supplementary material available at 10.1186/s12913-022-07516-2.

## Background

The assessment of responsiveness is now considered a key objective by health systems [1]. The concept was developed by the World Health Organization to assess the response to legitimate patient expectations for non-medical aspects of health care [2]. This includes people’s expectations of how they should be treated and in what environments and, on the other hand, people’s experiences when interacting with the health system regarding participation processes [3]. In this sense, Valentine et al. established three levels of responsiveness: the context where services are provided, users and providers that define the need for attention, and the individual care process [4].

On the other hand, Mirzoev et al. determined a conceptual framework in which people’s interactions and experiences are the basic components of the health system, so clarifying these expectations can help respond to people’s needs [5].

Unlike patient satisfaction, mainly focused on the clinical care received, responsiveness is oriented to the health system as a whole. It is expected that, if patient expectations are met, satisfaction with the received care, which has been related to both therapeutic compliance and clinical outcomes [6], will improve.

Primary care can be defined as “the provision of integrated and accessible health care services by clinicians who are accountable for addressing a large majority of personal health care needs, developing a sustained partnership with patients and practicing in the context of family and community” [7]. One of the main characteristics of primary care is universality and accessibility, facilitating continuity and coordination of care, and becoming the health service with more patients cared for [8]. Studies on the responsiveness of primary care have generally focused on specific population groups, as in the case of those developed by Kerssens et al. on a sample of fragile patients [9]. In other cases, research has been aimed at comparing public with private services, as well as urban services with rural ones [10].

A study on primary care in different European countries concluded that responsiveness is associated with the type of link the physician has with the health system and with health expenditure, so patients cared for by paid physicians via capitation positively value the attention received, and in countries with higher expenditure on health, a better assessment of the dignity and autonomy domains is recorded [11, 12]. In Spain, with mixed remuneration (salary and capitation), responsiveness was valued as poor [11].

With regard to mental health, the prevalence of mental disorders in the Spanish adult population has been estimated at 10.7% [13]. People with mental health problems frequently interact with primary care services. Thus, up to 58.8% of the health problems addressed in family medicine consultations correspond to mental illnesses, being anxiety (46.7%) and depression (41.7%) the most common [14]. These reach 47% in people over the age of 75, who are commonly associated with other comorbidities [15].

In Spain, a community model of care has been followed for people with mental health problems with the aim of providing comprehensive care, focusing on prevention and following the principles of accessibility, continuity, autonomy, and equity. That is why community mental health professionals work in a coordinated and interdisciplinary manner with primary care teams [16]. The Spanish mental health system is composed of a specialized and integrative network that supports primary care, and which is made up of salaried professionals and residential and intermediate centres in the community, where there are multidisciplinary clinical teams that provide services. In primary care, it is general practitioners who care for patients, establishing treatment or referring them to a specialized network. In recent years, some elements such as person-centered care, population-based improvements, user experience, and a look to the costs and care of the professional have been added to this model of community mental health. Progress has also been made in the development of assessment models, although a future national mental health strategy should incorporate the participation of patients and their families [17].

In addition, the stigma associated with mental illness and its treatment makes these patients more vulnerable and discourages individuals from getting proper mental health treatment [18]. So it is interesting to know their perception of primary care responsiveness and its associated factors in order to adapt the care provided to the needs of patients.

The objective of this study was to evaluate the responsiveness of the health system in primary care, both globally and for each of the domains, as valued by people with mental illness, with or without other chronic diseases, and to identify socioeconomic variables associated with poor responsiveness.

## Methods

Cross-sectional descriptive study through the use of a validated questionnaire.

All methods were carried out in accordance with relevant guidelines (STROBE) and regulations (COPE, ICMJE).

The study population consisted of patients over the age of 18, with at least one mental illness (no specific type of mental illness was included), in pharmacological and/or psychological treatment who, for any reason, had attended primary care consultations of the public health system in any of two Spanish regions (Aragon and La Rioja) in the 12 months prior to the start of the study. Aragon and La Rioja are two regions of northern Spain, which have a population density of 27.8 inhabitants/Km2 and 63.4 inhabitants/Km2 respectively.

Other inclusion criteria were not having cognitive impairment or receiving palliative care. To confirm the absence of cognitive impairment, the Mini-Mental State Examination was performed.

Consecutive sampling was performed for all patients meeting the inclusion criteria. The sample size was calculated considering that the proportion of patients reporting good responsiveness is a maximum of 53% [19]. With an accuracy of 5% and a 95% confidence interval, it was determined that 383 people should be included. Rating an expected 10% dropout rate, 426 people (213 from each region) had to be recruited.

Sources for data collection were the electronic medical history and interviews with the patients. Data were collected for all factors that could influence the assessment of the health system responsiveness, such as socioeconomic variables, self-perceived health status, and place of residence. The state of self-perceived health refers to the sociodemographic question: “*Generally speaking, how would you describe your current state of health?*”. This answer could range from Very good/good, normal, bad/very bad.

As an outcome variable, the assessment of the responsiveness of the global health system and for each of the domains was considered (see Supplemental Digital Content 1). To this end, the WHO “Multi-country Survey Study on Health and Health Systems Responsiveness (MCSS)” was used, which has been developed in 61 countries and validated in Spanish [19]. Seven of the eight domains (dignity, confidentiality, communication, autonomy, prompt attention, quality of basic amenities, and choice of provider) were considered, as the social consideration domain is only valued in patients admitted to hospital. These domains were measured on a Likert scale with five categories (very poor, poor, moderate, good, and very good), which were grouped for their analysis into “good” responsiveness (combining “good” and “very good”) and “poor” responsiveness (with “moderate”, “poor”, and “very poor” responses combined) (see Supplemental Digital Content 1). In addition, participants valued which domain was the most important to them.

Data were collected from July 2018 to December 2019 through 30-min interviews conducted by eight trained surveyors. Family physicians invited patients who met the inclusion criteria to participate in the survey. Those who accepted the invitation were referred to an interviewer who provided them with information about the characteristics and duration of the questionnaire and requested consent to participate in the study. Individual, structured interviews were conducted.

A pilot study was conducted to detect and address reliability issues in the data collection notebook.

The statistical analysis was performed with the SPSS software (Statistical Package for Social Sciences) V.24.0 (IBM SPSS). After cleaning up data, quantitative and qualitative variables were analysed using numerical and graphical statistics. To compare sociodemographic variables and responsiveness, contingency tables were made, and the Chi-squared test was used. OR and 95% confidence interval (95% CI) were calculated. A logistic regression model determined the association between geographic area and responsiveness, controlling the confounding variables.

### Ethical considerations

This protocol was approved by the Aragon Research Ethics Committee (Zaragoza, Spain), with number code PI17/194. All participants signed an informed consent prior to conducting the interviews.

## Results

### Characteristics of the participants

Four hundred twenty-nine people with mental illness were interviewed. 78.8% (*n* = 338) were women and 21.2% (*n* = 91) were men. The mean age was 62 years (CI 95%: 60.5–63.6). A 68% of the participants belonged to the region of Aragon, the 41.5% resided in a city of more than 50,000 inhabitants, and 38.9% had a bad or very bad state of self-perceived health.

In 80.7% of patients, there was an associated chronic disease: 35.4% had high blood pressure, 13.3% were diabetic, and 13.5% had a rheumatological disease.

The sample characteristics according to the presence/absence of chronic disease are presented in Table 1.

**Table 1:** Sociodemographic characteristics according to the presence of a chronic illness

	Patients with mental illness n (%) ***n*** = 83	Patients with mental illness and other chronic illness n (%) ***n*** = 346	p
**Sex**			0.63
Male	16 (19.3)	75 (21.7)	
**Age (years)**			0.001
< 60	66 (81.5)	127 (38)	
≥ 60	15 (18.5)	207 (62)	
**Marital status**			0.001
Single	18 (19.3)	42 (12.1)	
Separate	13 (15.7)	27 (7.8)	
Married/living as a couple	51 (61.4)	200 (57.8)	
Widow/er	3 (3.6)	77 (22.3)	
**Level of studies**			0.001
Basic	19 (22.9)	197 (56.9)	
Intermediate	50 (60.2)	117 (36.7)	
High	14 (16.9)	22 (6.4)	
**Area of residence**			0.07
< 10.000 Inhab.	35 (42.2)	102 (29.5)	
10.000 a 50.000 Inhab.	17 (20.5)	97 (28)	
> 50.000 Inhab.	31 (37.3)	147 (42.5)	
**Occupation**			0.001
Employed	23 (27.7)	53 (15.3)	
Unemployed	29 (34.9)	77 (22.3)	
Retiree/Disabled	31 (37.3)	216 (62.4)	
**Social status**			0.14
Low	40 (49.4)	132 (40.5)	
Intermediate-high	41 (55.6)	194 (59.5)	
**Level of income**			0.005
< 900 €/month	5 (7.5)	73 (25.8)	
≥ 900 €/month	62 (92.5)	210 (74.2)	
**Type of psychiatric illness**			0.32
Schizoaffective disorder	4 (4.8)	11 (3.2)	
Depressive disorder	28 (37.7)	147 (42.5)	
Anxiety disorder	39 (47)	130 (37.6)	
Other	12 (14.5)	58 (16.8)	
**Duration of psychiatric illness**			0.05
More than 10 years	21 (31.8)	129 (44.6)	
Less than or 10 years	45 (68.2)	160 (55.4)	
**Private insurance**			0.27
Yes	13 (15.7)	34 (9.8)	
**Attending to family physician**			0.90
Never	12 (14.5)	50 (14.5)	
1 to 4 times	67 (80.7)	283 (81.8)	
More than 4 times	4 (4.8)	13 (3.8)	
**Attending to PC nursing**			0.03
Never	59 (71.1)	193 (55.8)	
1 to 4 times	21 (25.3)	140 (40.5)	
More than 4 times	3 (3.6)	13 (3.8)	
**Attending to psychologist**			0.04
Never	71 (85.5)	323 (93.4)	
1 to 4 times	12 (14.5)	22 (6.4)	
More than 4 times	0 (0)	1 (0.3)	
**Health state**			0.08
Good/Very good	26 (31.3)	74 (21.4)	
Normal	32 (38.6)	130 (37.6)	
Bad/Very bad	25 (30.1)	142 (41)	

### Use of medical health services

In the 30 days prior to the interview, 81.6% of patients had visited the family physician and 37.5% had visited the community nurse. When they needed prompt attention, 45.7% said they had always obtained it.

The mean age of family physicians was 51.6 years (SD = 0.28) and 78.8% were women.

### Health system objectives

63.3% of participants believed that the most important goal of the health system was to improve health for all; for 61.1%, the second in importance was improving the treatment of people when receiving health care; and for 85.3% of the participants, the least important objective was that of the economic contribution that each must make to sustain the health system

### Primary care responsiveness

Overall responsiveness was good for 77.4% of patients (CI 95%: 73.2–81.1), being this probability higher in the domains: dignity 96.7% (CI 95%: 94.6–98), confidentiality 94.7% (CI 95%: 92.1–96.5), and communication 94.2% (CI 95%: 91.5–96).

Responsiveness was poor most often in the domains: autonomy 12.7% (CI 95%: 9.7–16.2), choice 13.2% (CI 95%: 9.9–17 4), prompt attention 25.8% (CI 95%: 21.8–30.2), and quality of basic amenities 50.9% (CI 95%: 46.2–55.6).

57.8% of patients who experienced poor responsiveness regarding prompt attention also showed poor overall responsiveness (*p* < 0.005)

**Fig. 1:**
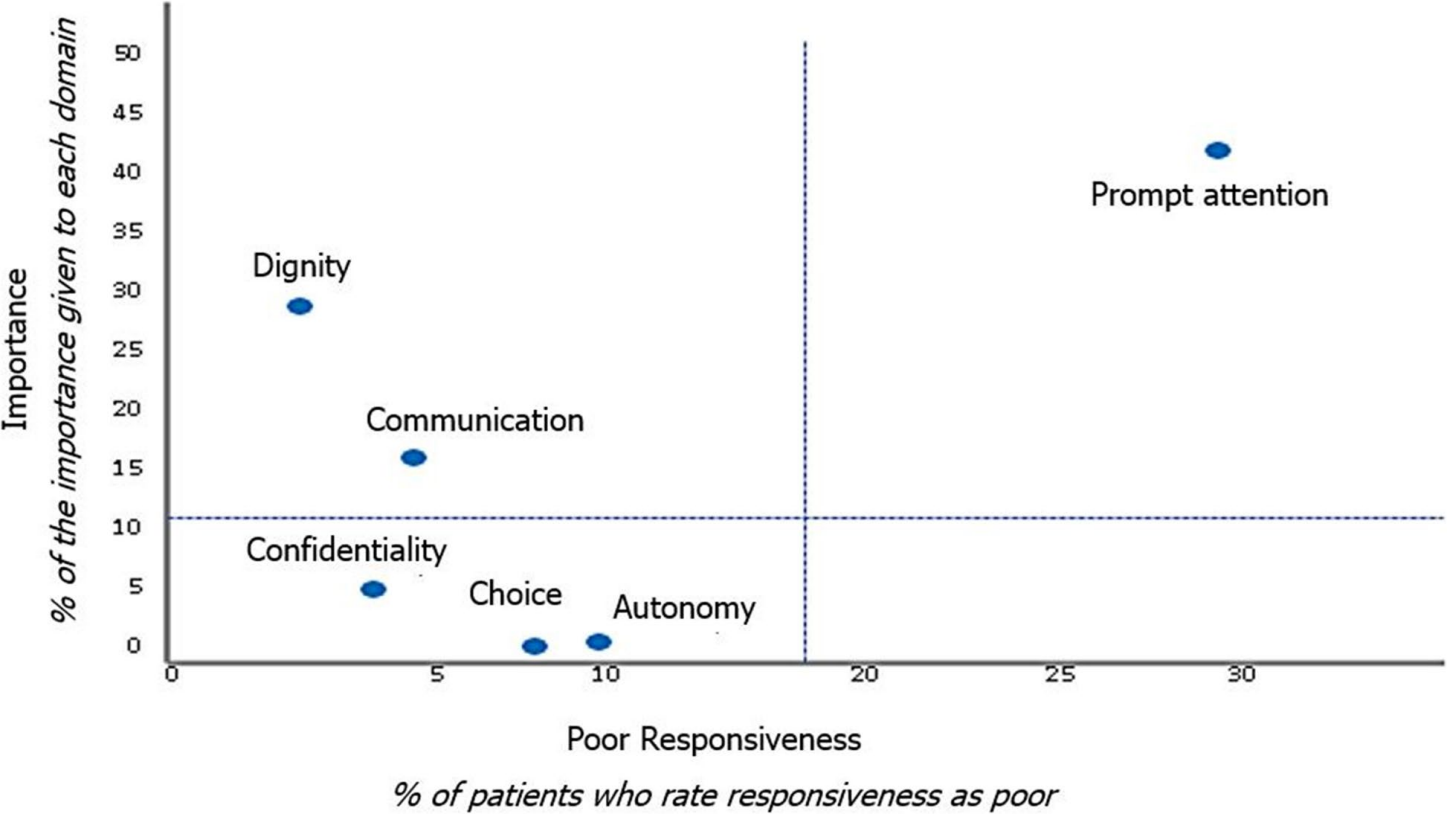
Relationship between the importance of the domains and responsiveness

The most valued domains by people with mental illness were prompt attention (42.4%), dignity (30.1%), and communication (17%), but only prompt attention scored high in importance and poor in responsiveness (Fig. 1).

Choice (10.7%) was the domain that was given the lowest value, along with autonomy (8.9%) and confidentiality (3.3%).

### Responsiveness, social status, and educational level

In 19.4% of patients with an income below 900 euros per month, responsiveness was poor for the autonomy domain, as compared to 11.3% (*p* = 0.06), and for 10.5% of them, confidentiality was poor versus 3.8% of them (*p* = 0.02). Among these, for those with an income of 900 or more euros per month, the probability of rating responsiveness as poor regarding confidentiality was multiplied by 3 (OR = 3; CI 95%: 1.1–7.8).

7.6% of those with a low level of studies showed poor responsiveness in confidentiality, as compared to 2.9% (*p* = 0.03), and 19.6% valued prompt attention as poor versus 32.2% (*p* = 0.003) (Table 2). Compared to those with a high level of studies, the likelihood of rating responsiveness as poor in confidentiality terms was multiplied by 2.7 (95% CI: 1.1–7.2) and by 0.5 (CI 95%: 0.3–0.8) as regards prompt attention (Table 3)

**Table 2:** Proportion of responsiveness according to socioeconomic level, level of studies, and place of residence

Poor responsiveness	Social status (Low)	Economic income (< 900 euros/month)	Level of studies (Low)	Size of place of residence (> 50.000 inhab.)
**Global**	22.7	25.6	21.8	24.7
**Dignity**	4.1	2.6	3.2	4.5
**Confidentiality**	6.5	10.5*	7.6*	3.9
**Communication**	6.4	7.7	6.5	5.1
**Autonomy**	14.5	19.4	13.2	14
**Choice**	15	10.3	15.3	10.7
**Prompt attention**	26.7	22.4	19.6*	38.8*
**Quality of amenities**	49.1	55.8	52.3	56.2

*p < 0.05

**Table 3:** Association between responsiveness and Sociodemographic variables. Logistic regression

	Place of residence Ref: < 50.000 Inhab. OR (CI 95%)	Social status Ref: Intermediate-high OR (CI 95%)	Economic income Ref: ≥ 900 Euros OR (CI 95%)	Level of studies Ref: intermediate-high OR (CI 95%)	Illness evolution Ref: ≤10 years OR (CI 95%)
**Global**	1.2 (0.8–1.9)	1 (0.6–1.6)	1.4 (0.8–2.4)	0.9 (0.5–1.4)	0.9 (0.6–1.6)
**Dignity**	1.9 (0.7–5.6)	1.6 (0.5–1.9)	0.9 (0.2–2.2)	0.9 (0.3–2.8)	0.8 (0.3–2.6)
**Confidentiality**	0.29 (0.24–1.53)	1.5 (0.6–3.6)	3 (1.1–7.8)*	2.7 (1.1–7.2)*	1.3 (0.6–3.4)
**Communication**	0.78 (0.34–1.8)	1.1 (0.5–2.6)	2.2 (0.8–6.2)	1.2 (0.5–2.8)	1.2 (0.5–2.9)
**Autonomy**	1.2 (0.7–2.2)	1.3 (0.7–2.3)	1.9 (0.9–3.8)	1.1 (0.6–1.9)	1.3 (0.7–2.5)
**Choice**	0.62 (0.32–1.2)	1.2 (0.6–2.4)	0.8 (0.3–2.1)	1.4 (0.7–2.8)	2.6 (1.2–5.4)*
**Prompt attention**	3 (1.9–4.8)*	1.5 (0.8–1.8)	0.9 (0.6–1.5)	0.5 (0.3–0.8)*	1.4 (0.9–2.3)
**Cleanliness**	2 (1.3–3.1)*	0.5 (0.3–0.9)*	1.2 (0.7–2.1)	0.7 (0.5–1.1)	0.8 (0.5–1.3)
**Amenities**	1.8 (1.2–2.7)*	0.7 (0.5–1.2)	1.4 (0.8–2.4)	0.8 (0.5–1.2)	0.8 (0.5–1.2)
**Quality of services**	1.4 (0.9–2.1)	1.2 (0.8–1.7)	1.2 (0.7–2.1)	1.1 (0.7–1.6)	0.7 (0.4–1)

*p < 0.05

### Responsiveness and health status

The presence of other chronic diseases was related to good responsiveness in all domains, except for the quality of basic amenities, where 51.9% rated it as poor, though the differences were not significant.

**Fig. 2:**
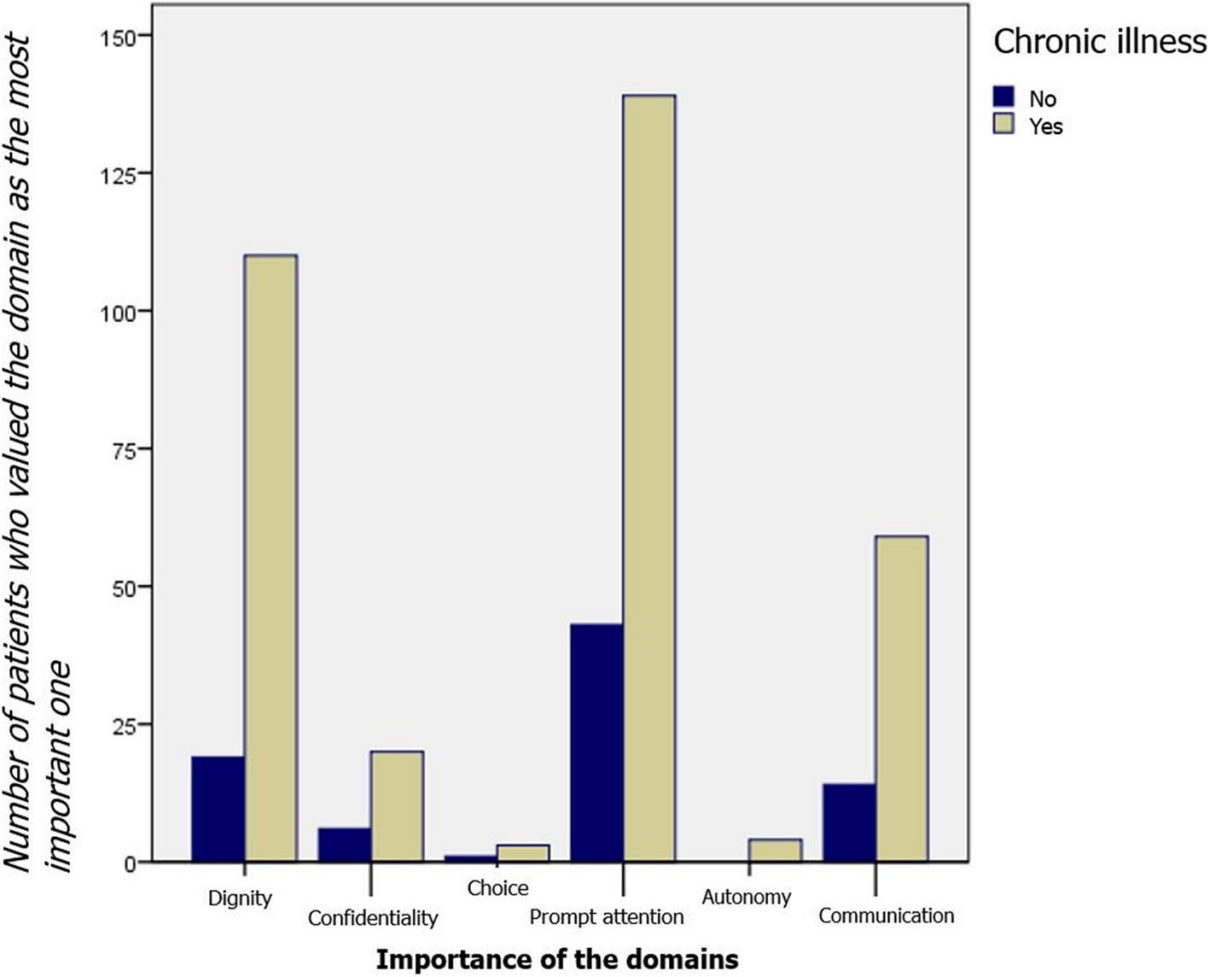
Representation of the importance of the domains according to the presence of chronic illness

People with mental illness and other chronic disease most importantly valued the domains of prompt attention (41.5%), dignity (32.8%), and communication (17.6%), albeit without statistical significance (Fig. 2).

An association was found between the health state and some domains. Among those who perceived their health state as bad or very bad, 35.8% rated responsiveness regarding prompt attention as poor, as also 5.4% as regards the dignity domain (*p* < 0.05).

In patients with a mental illness of more than 10 years of evolution, the likelihood of valuing responsiveness as poor in the choice domain was multiplied by 2.6 (Table 3).

The number of visits to the family physician was also related to some domains. 34.4% of patients who had attended the consultation between once and four times in the previous month scored poor when it came to cleanliness (p-0.009), and 11.5% in the choice domain (*p* = 0.04).

### Responsiveness and environment

38.8% of patients living in areas larger than 50,000 people valued responsiveness as poor in the prompt attention domain, versus 17.3% (*p* < 0.05). There were no significant differences for the other domains

Among these patients, for those residing in areas of less than 50,000 inhabitants, the likelihood of scoring responsiveness as poor was multiplied by 3 as regards prompt attention, by 2 when it came to cleanliness, and by 1.8 regarding basic amenities (*p* < 0.05).

In the multiple regression performed, the relationship between an income lower than 900 euros per month and poor responsiveness in confidentiality did not change when adjusting the size of the place of residence.

## Discussion

This study has assessed primary care responsiveness as valued by people with mental illness in two Spanish regions, and the factors associated with poor responsiveness.

For most patients, overall responsiveness was good, the highest percentages corresponding to domains that indicate respect for people: dignity, confidentiality, and communication.

Dignity was the domain most often valued as good, implying that people with mental illness perceive respectful treatment without stigmatisation from primary care professionals. Confidentiality was the second in frequency, indicating that patients are confident in that information about their disease will be maintained private. These findings are also described in studies conducted on people with mental illness outside the scope of primary care [20, 21]. Confidentiality relates to the preservation of personal data, and this is an important aspect in these patients as, when this is not guaranteed, there may be a refusal to share the information [22]. In the present study, most of the participants valued this dimension as good by generally indicating a high degree of trust in the family physicians who assist them.

Often, responsiveness was rated as poor in the autonomy domain and in those dealing with the provision of services such as choice, prompt attention, and quality of basic amenities. Other studies find similar results, as in Iran’s mental health services [20], where prompt attention, quality of basic amenities, and autonomy were the domains with the worst valued responsiveness, or in Germany [21], where this was the case for the quality of basic amenities and autonomy domains.

It has been suggested that some changes in the interaction between healthcare professionals and patients, such as maintaining an empathetic relationship, may serve as a tool to increase their experience of participation and thus promote their autonomy [23]. People with mental illness want more active participation in treatments and service planning [24], therefore primary care is an optimal location for mental health services because of the possibilities for continuity of care and therapeutic relationships, which is of utmost importance to patients [25]. That is why promoting certain behaviours from institutions and the professionals working in them could contribute to reducing overprotecting attitudes in treating these patients.

The perception of poor responsiveness in prompt attention can be explained by the increased demand for primary care that also increases the time to be assisted, as suggested by Liu et al. [26] in their study. In addition, this is the domain that scored highest in importance and poor responsiveness.

Unlike Bramenfeld et al.’s [21] findings in outpatient care for patients with mental illness, where autonomy and prompt attention were considered the most important domains, in the present study, these domains are prompt attention, dignity, and communication.

In patients with a poor health state, responsiveness was valued as poor in the domains of prompt attention and dignity. Unlike this, in Forouzan et al.’s study, experience with dignity in mentally ill patients who had been hospitalised was poor for those with a good health state [20].

It has been found that responsiveness is related to the duration of the mental illness, so it is poor in the choice domain for patients with a disease of more than 10 years of evolution. This can be explained because, over time, the ability to choose new services is reduced. However, in Bramesfeld’s study al [21]., those who had long suffered the disease perceived responsiveness as good, which is attributed to the reduction in expectations of these patients.

For patients with an income lower than 900 euros (€) per month, responsiveness was poor in the autonomy and confidentiality domains, and also in confidentiality for those with a low level of studies. However, among these, the likelihood that the responsiveness in prompt attention was poor was reduced by half. These findings are consistent with those from other studies [21].

Institutions should include the necessary actions to improve care in the mental health plans of people with fewer economic resources and a low level of education. Thus, Aragon’s mental health strategy, being one of the regions where the study has been carried out, includes procedures and resources to ensure access to services and continuity of care [27].

Unlike the Gabrani J. et al.’s [10] study, responsiveness in prompt attention was worse valued in urban areas. To try to alleviate this situation, measures such as improving times organisation and an adequate allocation of tasks among primary care professionals, as well as carrying out patient health education interventions [28], should be implemented.

In the present study, only 7 of the 8 responsiveness dimensions defined by WHO were used, as one of them was not applicable to primary care services. Possible information biases have been minimised through questionnaire piloting and surveyors’ training.

## Conclusions

People with mental illness perceive good responsiveness of the primary care system in terms of dignity, confidentiality, and communication. Prompt attention should be prioritised as the domain of greatest importance and worst valuation through the implementation of organisational measures in health centres to reduce waiting times, especially in urban areas.

It is necessary to give patients more participation opportunities in decision-making and provide confidence in the guarantee of privacy of their personal data, mainly in those with fewer economic resources and low educational level.

## Supplementary Information

**Additional file 1: Supplemental Digital Content 1.** Definition of the domains (WHO).

## Data Availability

The dataset supporting the conclusions of this article is included within the article (and its additional file).

## Abbreviations

WHO: World Health Organization
MCSS: Multi-country Survey Study on Health and Health Systems Responsiveness
SPSS: Statistical Package for Social Sciences
OR: Odds Ratio
CI: Confidence Interval
SD: Standard Deviation
