# Supplementary material for: Evaluation of primary care responsiveness by people with mental illness in Spain
Source: BMC Health Serv Res. 2022 Jan 31;22:133. doi: 10.1186/s12913-022-07516-2 (PMC8805273; doi:10.1186/s12913-022-07516-2)
Supplement: Supplementary file 1 — Additional file 1: Supplemental Digital Content 1. Definition of the domains (WHO). [file 12913_2022_7516_MOESM1_ESM.docx]

**Supplemental Digital Content 1.** Definition of the domains (WHO)

| Respect for people | Dignity | Being shown respect by health professionals. Having physical examinations conducted in privacy. |
| --- | --- | --- |
|  | Confidentiality | Having medical history kept confidential. Having consultations with health providers done so that privacy is maintained. |
|  | Communication | Carefully listening to patients. Explaining things so that they can be understood. Close dialogue between patient and provider. |
|  | Autonomy | Involving the patient in decision-making on their care or treatment. Having the provider ask permission before starting treatments or tests. |
| Client focus | Prompt Attention | Fast care in emergencies. Short waiting times for appointments, consultations, tests, treatment, and hospital admission. |
|  | Quality of Basic Amenities | Enough space, seating and fresh air in the waiting room, clean facilities (including clean toilets), with adequate furniture. |
|  | Choice of Care Provider | Being able to choose health provider and services. |
